# Outcomes of Adults with Severe Aortic Stenosis Undergoing Urgent or Emergent vs. Elective Transcatheter Aortic Valve Replacement Within an Integrated Health Care Delivery System

**DOI:** 10.1016/j.shj.2023.100166

**Published:** 2023-03-21

**Authors:** Justin J. Slade, Andrew P. Ambrosy, Thomas K. Leong, Sue Hee Sung, Elisha A. Garcia, Ivy A. Ku, Matthew D. Solomon, Edward J. McNulty, Andrew N. Rassi, David C. Lange, Femi Philip, Alan S. Go, Jacob M. Mishell

**Affiliations:** a Department of Cardiology, Kaiser Permanente San Francisco Medical Center, San Francisco, California, USA; b Division of Research, Kaiser Permanente Northern California, Oakland, California, USA; c Department of Cardiology, Kaiser Permanente Santa Clara Medical Center, Santa Clara, California, USA; d Department of Cardiology, Kaiser Permanente Sacramento Medical Center, Sacramento, California, USA; e Department of Health Systems Science, Kaiser Permanente Bernard J. Tyson School of Medicine, Pasadena, California, USA; f Departments of Epidemiology, Biostatistics and Medicine, University of California, San Francisco, San Francisco, California, USA; g Department of Medicine, Stanford University, Palo Alto, California, USA

**Keywords:** Aortic stenosis, Quality-of-life, TAVR outcomes, Urgent/emergent

## Abstract

**Background:**

Transcatheter aortic valve replacement (TAVR) may be used to urgently or emergently treat severe aortic stenosis, but outcomes for this high-risk population have not been well-characterized. We sought to describe the incidence, clinical characteristics, and outcomes of patients undergoing urgent or emergent vs. elective TAVR.

**Methods:**

We identified all adults who received TAVR for primary aortic stenosis between 2013 and 2019 within an integrated health care delivery system in Northern California. Elective or urgent/emergent procedure status was based on standard Society of Thoracic Surgeons definitions. Data were obtained from electronic health records, the Society of Thoracic Surgeons-American College of Cardiology Transcatheter Valve Therapy Registry, and state/national reporting databases. Logistic regression and Cox proportional hazard models were performed.

**Results:**

Among 1564 eligible adults that underwent TAVR, 81 (5.2%) were classified as urgent/emergent. These patients were more likely to have heart failure (63.0% vs. 47.4%), reduced left ventricular ejection fraction (21.0% vs. 11.8%), or a prior aortic valve balloon valvuloplasty (13.6% vs. 5.0%) and experienced higher unadjusted rates of 30-day and 1-year morbidity and mortality. Urgent/emergent TAVR status was independently associated with non-improved quality of life at 30-days (hazard ratio, 4.87; *p* < 0.01) and acute kidney injury within 1-year post-TAVR (hazard ratio, 2.11; *p* = 0.01). There was not a significant difference in adjusted 1-year mortality with urgent/emergent TAVR.

**Conclusions:**

Urgent/emergent TAVR status was uncommon and associated with high-risk clinical features and higher unadjusted rates of short- and long-term morbidity and mortality. Procedure status may be useful to identify patients less likely to experience significant short term improvement in health-related quality of life post-TAVR.

## Highlights

• Patients undergoing urgent or emergent vs. elective transcatheter aortic valve replacement (TAVR) were more likely to have heart failure, a reduced left ventricular ejection fraction, acute myocardial infarction or unstable angina, or a prior aortic valve balloon valvuloplasty.
• Similar, high rates of non-cardiovascular comorbidities (diabetes, chronic kidney disease, chronic lung disease, chronic liver disease) are observed in the urgent/emergent and elective TAVR populations.
• A higher unadjusted rate of all-cause 1-year mortality was observed in those receiving urgent/emergent vs. elective TAVR (10.7 vs. 3.6 per 100 person-years).
• Urgent/emergent TAVR status is independently associated with non-improved quality of life at 30 days and a higher rate of acute kidney injury within 1 year post-TAVR.

## Introduction

Since the U.S. Food and Drug Administration approved the Edwards SAPIEN balloon expandable valve for use in inoperable patients with severe aortic stenosis in 2011, there has been exponential growth in the use of transcatheter aortic valve replacement (TAVR). In 2019, 79,991 TAVR procedures were performed nationally, exceeding surgical aortic valve replacement for the first time.^1^ While typically an elective procedure, TAVR performance in non-elective scenarios has also expanded in the U.S, with 8.4% occurring in urgent, emergent, or salvage cases in 2019. Despite this trend, outcomes for individuals undergoing urgent or emergent TAVR have not been fully characterized. The pivotal randomized controlled trials supporting the use of TAVR defined their populations based on surgical risk and did not compare relative outcomes based on the acuity status of patient presentation.^2^, ^3^, ^4^, ^5^, ^6^, ^7^

The Society of Thoracic Surgeons-American College of Cardiology Transcatheter Valve Therapy Registry (STS/ACC TVT Registry) has captured acuity status for patients undergoing TAVR at sites within the U.S., defining urgent TAVR as a “procedure required during the same hospitalization to minimize chance of further clinical deterioration. Examples include but are not limited to: worsening, sudden chest pain, heart failure, acute myocardial infarction, anatomy, intra-aortic balloon pump, unstable angina with intravenous nitroglycerin, or rest angina.”^8^ The registry defines those undergoing emergent TAVR as having “ongoing, refractory (difficult, complicated, and/or unmanageable) unrelenting cardiac compromise, with or without hemodynamic instability, and not responsive to any form of therapy except cardiac surgery.” An analysis of this registry’s data compared 40,042 patients that underwent TAVR between November 2011 and December 2016 and found that patients undergoing urgent or emergent TAVR had higher rates of 30-day (8.7% vs. 4.3%) and 1-year (29.1% vs. 17.5%) mortality as well as acute kidney injury (AKI) and/or new dialysis (8.2% vs. 4.2%) during their index hospitalization in comparison to elective cases.^9^ Several smaller observational studies, including an analysis of a multicenter Japanese registry, have yielded similar findings with respect to 30-day and 1-year mortality as well as AKI.^10^, ^11^, ^12^ Additionally, urgent TAVR was associated with a higher rate of 30-day all-cause readmission (15.5% vs. 9.5%) relative to elective TAVR in a retrospective cohort study from the Nationwide Readmission Database.^13^ To date, the range of baseline characteristics and outcomes for these patients has been limited by the data fields tracked within these registries, which may lack some data elements that can be captured within a comprehensive electronic health record (EHR) system. Notably, potential differences in quality of life and other post-hospitalization outcomes including emergency department visits, AKI, and cardiovascular events following urgent vs. elective TAVR have not been well studied.

Thus, the primary objectives of this study were to further characterize the population of patients undergoing urgent or emergent TAVR and to compare contemporary 30-day and 1-year outcomes with those from the elective TAVR population within an integrated health care delivery system.

## Methods

### Source Population

The study population was derived from the membership of Kaiser Permanente Northern California (KPNC), an integrated health care delivery system currently caring for >4.5 million members in Northern California. KPNC provides comprehensive care tracked through EHR systems, including diagnoses, procedures, health care utilization, laboratory results, and medication dispensing. In addition to the KPNC EHR, the STS/ACC TVT Registry was used to identify additional data elements including presentation, detailed pre-procedure transthoracic echocardiogram results, procedure information, and health-related quality of life (i.e., Kansas City Cardiomyopathy Questionnaire [KCCQ]). Membership is highly representative of the local and statewide population with respect to age, gender, race/ethnicity, and socioeconomic status.^14^, ^15^, ^16^ This study was approved by the KPNC Institutional Review Board, and a waiver of informed consent was obtained as this is a retrospective, data-only study.

### Study Sample

We identified all KPNC members aged ≥18 years who received TAVR for primary severe aortic stenosis between January 1, 2013 and December 31, 2019 that were captured in the STS/ACC TVT Registry data. We excluded patients who had ≤12 months of health plan membership before TAVR to ensure availability of baseline data. We determined patients’ status at presentation using data entered in the STS/ACC TVT Registry and categorized each patient as either undergoing an urgent or emergent TAVR or an elective TAVR. This determination was made by the primary TAVR operator at the time of the procedure. We excluded 1 patient without documented classification of TAVR status from this analysis.

### Covariates

We ascertained baseline sociodemographics and clinical characteristics using *International Classification of Diseases*-9/10 and Current Procedural Terminology diagnostic or procedure codes, laboratory results, vital signs, or specific therapies received based on validated algorithms and approaches, as well as from data entered in the STS/ACC TVT Registry. These included demographic characteristics, cardiovascular and other medical history, anatomical measurements, prior laboratory results, prior medical utilization, and baseline quality of life. A full list of covariates is available upon request.

### Outcomes

We identified the following patient-centered and clinical outcomes of interest: improvement in quality of life, hospitalized AKI, ED visits, hospitalizations, heart failure (HF)-ED visits, HF-related hospitalizations, all-cause death, and a composite of cardiovascular events. We measured improvement of quality of life at 30 days and at 1-year after the date on which the patient received TAVR and identified all other outcomes on a time-to-first event basis from the index procedure date.

We defined improvement in quality of life as a 10-point improvement from baseline or having a KCCQ of at least 60 points at follow-up. Baseline quality of life was assessed prior to the procedure with follow-up measurements at 30-days and 1-year. We identified all-cause death from member proxy reporting, deaths identified during a hospitalization or ED visit from EHR and billing claims data, Social Security Administration vital status files and state death certificates.

We comprehensively identified ED visits and hospitalizations from EHR and billing claims data and classified each utilization episode as HF-related or not HF-related using previously validated approaches.^17^ Specifically, we classified ED visits as HF-related if they included a diagnosis code for HF using ICD Ninth (ICD-9) or Tenth (ICD-10) editions, and hospitalizations as HF-related if they included a principal discharge diagnosis code for HF (codes available on request).

We identified AKI during hospitalization using the serum creatinine-based Kidney Disease: Improving Global Outcomes (KDIGO) definition of either a 50% relative increase in serum creatinine compared to a prior outpatient measurement within 7 to 365 days preadmission or an absolute increase of 0.3 mg/dL over 48 ​hours during hospitalization.^18^

We also created a composite outcome of cardiovascular events, defined as any occurrence of hospitalization for HF, myocardial infarction, ischemic stroke, endocarditis, new-onset atrial fibrillation, or vascular complications. All conditions were identified using ICD-9 and ICD-10 diagnosis codes from EHR data, as well as reports of post-procedure complications from the STS/ACC TVT Registry data. Follow-up occurred through December 31, 2020 with censoring at health plan disenrollment, death, or end of study follow-up.

### Statistical Approach

Analyses were conducted using SAS 9.4 (SAS Analytics Software, Cary, North Carolina). We compared baseline characteristics by TAVR status using *t* tests for continuous variables and chi-squared tests for categorical variables. We identified all outcomes on a time-to-first event basis, calculated incidence rates (per 100 person-years) of outcomes at 30 and 365 days and compared rates by TAVR procedure status using a log-rank test. To assess the association of TAVR status with the study outcomes, we conducted logistic regression for the quality of life outcome and Cox proportional hazards models for all other outcomes, using a backward covariate selection approach starting with all potential covariates and a threshold for statistical significance of *p* < 0.05 for inclusion in the model.

## Results

### Cohort Assembly and Baseline Characteristics

**Figure 1:**
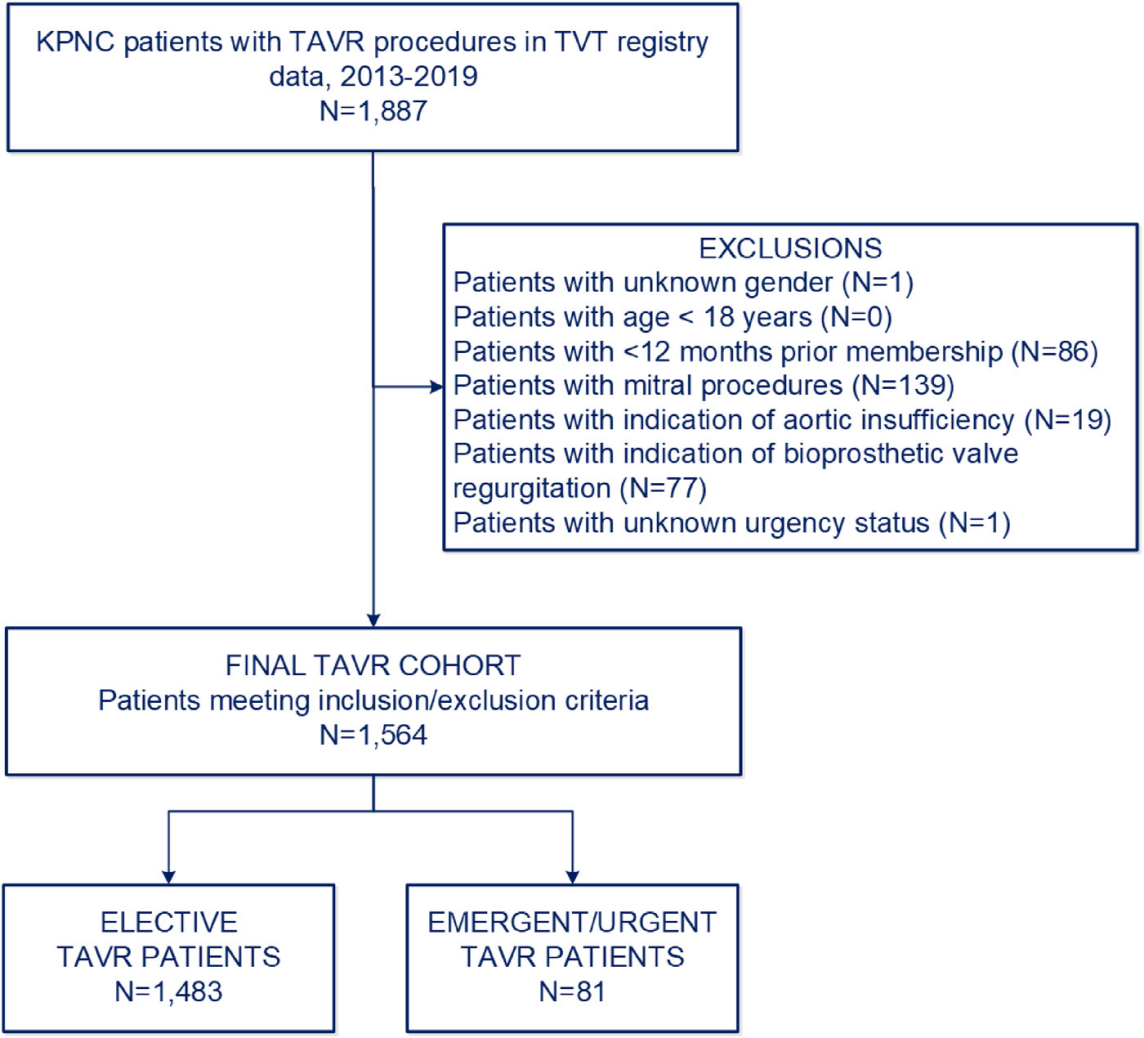
Assembly of the transcatheter aortic valve replacement (TAVR) study cohort. Abbreviations: KPNC, Kaiser Permanente Northern California; TVT, Transcatheter Valve Therapy.

During the study period, we initially identified 1887 patients from the STS/ACC TVT registry. Among 1564 eligible adults that underwent TAVR, 1483 (94.8%) were classified as elective and 81 (5.2%) were classified as urgent or emergent (Figure 1).

The urgent/emergent and elective TAVR cohorts were similar in age and gender (Table 1). Baseline quality of life was significantly worse in the urgent/emergent cohort, with a mean KCCQ score of 35.8 vs. 48.1 in the elective group (*p* < 0.001). Those undergoing urgent or emergent TAVR were more likely to have a prior aortic valve balloon valvuloplasty (13.6% vs. 5.0%), HF (63.0% vs. 47.4%), reduced left ventricular ejection fraction (21.0% vs. 11.8%), and prior acute myocardial infarction or history of unstable angina (24.7% vs. 10.9%). Although a high burden of diabetes, chronic kidney disease, and chronic lung disease was observed in the urgent/emergent TAVR group, there was no significant difference in prevalence of these and other major non-cardiac comorbidities compared to the elective TAVR group. In addition, there were similar rates of cerebrovascular and peripheral arterial disease between groups.

**Table 1:** Baseline characteristics overall and stratified by transcatheter aortic valve replacement procedure acuity status

Baseline characteristic	Overall (n = 1564)	Elective (n = 1483)	Urgent/emergent (n = 81)	*p*
Mean (SD) age, y	81.0 (8.2)	81.0 (8.2)	81.3 (8.2)	0.72
Women	679 (43.4)	651 (43.9)	28 (34.6)	0.10
Race/ethnicity				
White	1260 (80.6)	1203 (81.1)	57 (70.4)	
Black	51 (3.3)	50 (3.4)	1 (1.2)	
Asian or Pacific Islander	93 (5.9)	87 (5.9)	6 (7.4)	
Hispanic	134 (8.6)	121 (8.2)	13 (16.0)	
Other	26 (1.7)	22 (1.5)	4 (4.9)	
Cardiac History/comorbidities				
STS PROM				<0.05
<3%	344 (22.0)	331 (22.3)	13 (16.1)	
3%-8%	969 (62.0)	921 (62.1)	48 (59.3)	
>8%	201 (12.9)	188 (12.7)	13 (16.1)	
Missing	50 (3.2)	43 (2.9)	7 (8.6)	
Mean KCCQ-12 score (SD)	47.7 (23.5)	48.1 (23.5)	35.8 (20.6)	<0.001
Prior cardiac surgery	287 (18.4)	269 (18.1)	18 (22.2)	0.36
Prior balloon aortic valvuloplasty	85 (5.4)	74 (5.0)	11 (13.6)	<0.001
Prior heart failure	754 (48.2)	703 (47.4)	51 (63.0)	<0.01
Prior pacemaker	126 (8.1)	120 (8.1)	6 (7.4)	0.83
Reduced LVEF	192 (12.3)	175 (11.8)	17 (21.0)	<0.05
NYHA Class III or IV	1032 (66.0)	973 (65.6)	59 (72.8)	0.18
Acute MI or unstable angina	181 (11.6)	161 (10.9)	20 (24.7)	<0.001
Coronary revascularization	638 (40.8)	608 (41.0)	30 (37.0)	0.48
Atrial Fibrillation/flutter	609 (38.9)	573 (38.6)	36 (44.4)	0.30
Cerebrovascular disease	153 (9.8)	143 (9.6)	10 (12.3)	0.43
Peripheral artery disease	468 (29.9)	442 (29.8)	26 (32.1)	0.66
Non-cardiac comorbidities				
Anemia	70.9 (46.3)	670 (45.2)	39 (48.1)	0.60
Diabetes	620 (39.6)	588 (39.6)	32 (39.5)	0.98
Chronic kidney disease	652 (41.7)	618 (41.7)	34 (42.0)	0.96
Receiving chronic dialysis	63 (4.0)	59 (4.0)	4 (4.9)	0.67
Chronic lung disease	737 (47.1)	692 (46.7)	45 (55.6)	0.12
Chronic liver disease	224 (14.3)	214 (14.4)	10 (12.3)	0.60
Mean five-meter walk score (SD)	6.8 (2.2)	6.8 (2.3)	6.9 (1.8)	0.22
Procedural characteristics				
Degenerative AV etiology	1537 (98.3)	1456 (98.2)	81 (100.0)	0.22
Bicuspid AV	78 (5.0)	70 (4.7)	8 (9.9)	<0.05
Moderate/severe AI	184 (11.8)	176 (11.9)	8 (9.9)	0.59
Concurrent PCI	6 (0.4)	4 (0.3)	2 (2.5)	<0.01
Hybrid OR	1363 (87.1)	1301 (87.7)	62 (76.5)	<0.01
Mechanical support	8 (0.5)	8 (0.5)	0 (0.0)	0.51
General anesthesia	728 (46.5)	696 (46.9)	32 (39.5)	0.19
Transfemoral access	1446 (92.5)	1372 (92.5)	74 (91.4)	0.70
Post-procedure evidence of paravalvular leak	271 (17.3)	255 (17.2)	16 (19.8)	0.55
Procedure/device complications	121 (7.7)	113 (7.6)	8 (9.9)	0.46
Discharge to home	1451 (92.8)	1386 (93.5)	65 (80.3)	<0.001

AI, aortic insufficiency; AV, aortic valve; KCCQ-12, Kansas City Cardiomyopathy Questionnaire-12; LVEF, left ventricular ejection fraction; MI, myocardial infarction; n, number; NYHA, New York Heart Association; OR, operating room; PCI, percutaneous coronary intervention; STS PROM = Society of Thoracic Surgeons Predicted Risk of Mortality.

Urgent/emergent TAVR was performed more frequently in patients with bicuspid aortic valves (9.9% vs. 4.7%) and in those requiring concurrent percutaneous coronary intervention (2.5% vs. 0.3%). There were similar rates of moderate or severe aortic insufficiency in both groups (9.9% vs. 11.9%). There was no significant difference in rates of transfemoral access (91.4% vs. 92.5%) or mechanical circulatory support (0.0% vs. 0.5%).

### Outcomes of Interest

**Figure 2:**
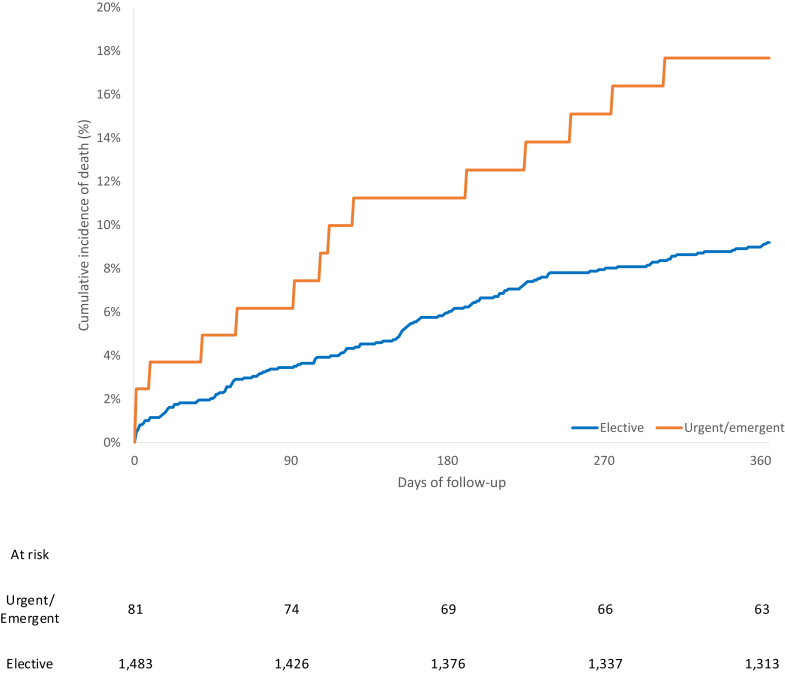
Incidence of death over time in elective vs. urgent/emergent transcatheter aortic valve replacement patients.

Patients undergoing urgent/emergent TAVR experienced higher unadjusted rates of death at 30 days (2.3 vs. 0.7 per 100 person-years) and 1 year (10.7 vs. 3.6 per 100 person-years) relative to those having elective TAVR (Table 2, Figure 2). There were lower rates of all-cause and HF hospitalization in the urgent/emergent TAVR group at 30 days, yet higher rates of all-cause and HF related ED visits at 30 days and 1 year. Vascular complications were more common in the urgent/emergent TAVR group, but no significant difference was identified between groups with respect to stroke, myocardial infarction, major bleeding, or endocarditis (Table 3).

**Table 2:** Unadjusted event rates and adjusted associations by procedure status for outcomes of interest

Outcomes	Unadjusted event rates			Adjusted associations	
	Overall (n = 1564) Rate per 100 person-y (95% CI)	Elective (n = 1483) Rate per 100 person-y (95% CI)	Urgent/Emergent (n = 81) Rate per 100 person-y (95% CI)	Urgent/Emergent vs. Elective (Ref) HR (95% CI)	*p*
All-cause death, 30 d	0.8 (0.5-1.1)	0.7 (0.5-1.1)	2.3 (0.7-7.1)	1.92 (0.29-12.59)	0.50
All-cause death, 1 y	3.9 (3.3-4.5)	3.6 (3.1-4.3)	10.7 (6.3–18.0)	1.76 (0.86-3.62)	0.12
Cardiovascular events, 30 d	149.1 (128.5-173.0)	144.3 (123.6-168.5)	241.1 (142.8-407.1)	1.10 (0.66–1.86)	0.71
Cardiovascular events, 1 y	29.3 (26.4-32.5)	29.0 (26.1-32.3)	34.0 (21.9-52.7)	0.95 (0.61-1.50)	0.84
All-cause hospitalization, 30 d	77.5 (63.6-94.3)	78.4 (64.1-95.8)	60.9 (22.9-162.3)	0.69 (0.25-1.92)	0.48
All-cause hospitalization, 1 y	44.9 (41.3-48.9)	44.9 (41.2-49.0)	45.3 (31.1-66.1)	0.82 (0.53-1.28)	0.39
HF hospitalization, 30 d	15.4 (9.9-23.8)	16.2 (10.4-25.1)	0.0 (0.0-0.0)	*no events*	-
HF hospitalization, 1 y	6.5 (5.3-8.0)	6.7 (5.5-8.3)	2.9 (0.7-11.5)	0.28 (0.06-1.28)	0.10
All-cause ED visit, 30 d	183.3 (160.7-209.1)	182.7 (159.6-209.1)	195.7 (111.1-344.5)	0.89 (0.49-1.64)	0.71
All-cause ED visit, 1 y	86.7 (80.9-92.8)	86.0 (80.1-92.2)	101.0 (75.2-135.7)	1.06 (0.77-1.47)	0.71
HF-related ED visit, 30 d	77.4 (63.5-94.3)	75.6 (61.6-92.9)	110.8 (52.8-232.5)	0.64 (0.25-1.67)	0.36
HF-related ED visit, 1 y	30.5 (27.6-33.8)	29.8 (26.8-33.1)	46.0 (31.1-68.0)	0.94 (0.57-1.55)	0.82
AKI, 30 d	18.5 (12.4-27.5)	18.6 (12.4-28.0)	15.1 (2.1-107.0)	0.46 (0.05-4.14)	0.49
AKI, 1 y	13.6 (11.7-15.6)	13.0 (11.2-15.1)	24.7 (15.1-40.3)	1.85 (1.04-3.29)	0.04
	**Proportion (95% CI)**				
No improvement in QoL, 30 d∗	8.4% (6.7%-10.1%)	8.1% (6.4%-9.7%)	16.7% (5.4%-27.9%)	6.92 (2.09-22.91)	<0.01
No improvement in QoL, 1 y∗	18.2% (16.0%-20.4%)	17.8% (15.5%-20.0%)	28.3% (15.3%-41.3%)	1.49 (0.60-3.70)	0.39

AKI, acute kidney; ED, emergency department; HF, heart failure; HR, hazard ratio; N, number; QoL, quality of life.

∗ Defined as a 10-point improvement from baseline or having a Kansas City Cardiomyopathy Questionnaire of at least 60 points at follow-up.

**Table 3:** Unadjusted event rates by procedure status at 1-year for additional outcomes

Outcomes	Overall (n = 1564) Rate per 100 person-y (95% CI)	Elective (n = 1483) Rate per 100 person-y (95% CI)	Urgent/emergent (n = 81) Rate per 100 person-y (95% CI)	*p*
Acute MI	1.88 (1.29-2.74)	1.98 (1.36-2.88)	0.00 (0.00-0.00)	N/A
New atrial fibrillation	10.04 (8.48-11.87)	9.75 (8.19-11.61)	15.85 (8.53-29.45)	0.07
Stroke/TIA	6.08 (4.91-7.52)	6.17 (4.97-7.66)	4.35 (1.40-13.50)	0.28
Major bleeding, follow-up	7.03 (5.77-8.57)	7.10 (5.80-8.69)	5.79 (2.17-15.42)	0.34
Vascular complications	3.11 (2.31-4.18)	2.82 (2.05-3.87)	9.02 (4.05-20.07)	0.004
Endocarditis	2.79 (2.04-3.80)	2.71 (1.96-3.74)	4.33 (1.40-13.41)	0.22

MI, myocardial infarction; N, number; TIA, transient ischemic attack.

### Multivariable Association of Acuity Status With Outcomes

In our multivariable analysis, urgent/emergent TAVR status was not independently associated with all-cause death at 30 days or at 1 year. With regards to 30-day and 1-year all-cause and HF-related ED visits and hospitalizations, there were no significant adjusted differences between groups. Similarly, there was no significant adjusted difference based on TAVR acuity in a composite of cardiovascular events at 30 days (hazard ratio [HR], 1.10; *p* = 0.71) and 1 year (HR, 0.95; *p* = 0.84). Urgent/emergent TAVR status was independently associated with a nearly 2-fold higher adjusted risk of hospitalized AKI within 1 year (HR, 1.85; *p* = 0.04). While the majority of patients in both groups experienced improved quality of life at 30 days, non-improved quality of life was more often observed in the urgent/emergent TAVR group (HR, 6.92; *p* < 0.01). There was no longer a significant between-group difference in quality of life at 1-year (HR, 1.49; *p* = 0.39).

## Discussion

In this community-based cohort of adults receiving TAVR, we found that urgent or emergent status was uncommon, accounting for approximately 5% of all TAVR procedures performed. These cases were associated with higher baseline rates of risk-enhancing clinical features including known HF, prior acute myocardial infarction, bicuspid aortic valve anatomy, and prior aortic valve balloon valvuloplasty. The urgent or emergent TAVR group experienced higher unadjusted rates of death, vascular complications, hospitalized AKI, and HF-related ED visits. Finally, urgent or emergent TAVR status was an independent clinical predictor of non-improved quality of life at 30 days and AKI requiring hospitalization at 1 year.

The rate of urgent or emergent TAVR in our population (5.2%) was lower than the rate reported from the broader STS/ACC TVT registry (8.9%) since 2011. This difference may be attributable to several factors. First, as the primary operator caring for a patient makes the determination of TAVR acuity before entry within the STS/ACC TVT registry, there is potential for intra- and inter-observer variability with regards to the application of registry definitions for urgent/emergent TAVR status. Second, there may be innate mechanisms within our fully integrated health care delivery system that reduce the frequency of patients requiring TAVR on a non-elective basis. For example, referral and evaluation patterns may be more expeditious in this setting relative to others. Variable payment incentives for inpatient vs. elective procedures relative to those in other practice models may also play a role. Third, it is possible that the expansion in TAVR indications towards lower risk patients coupled with higher procedural volume and operator comfort over the course of our study period has yielded a lower percentage of patients requiring TAVR in the urgent or emergent setting. While our community-based study was not powered to identify temporal trends over the study period, data from the TVT registry suggest that the rate of patients receiving urgent/emergent/salvage TAVR at the national level has remained relatively stable from 2014 through 2019 (8.86% vs. 8.44%).^1^ Finally, our lower observed prevalence of urgent/emergent TAVR may be due to the exclusion of failed bioprosthetic aortic valve and primary aortic insufficiency indications in this study. It has been demonstrated from STS/ACC TVT registry data that valve-in-valve TAVR performance makes up a greater percentage of urgent/emergent relative to elective TAVRs due to more acute clinical presentations in this high-risk cohort.^9^^,^^19^, ^20^, ^21^

Patients undergoing urgent/emergent TAVR had a higher unadjusted rate of death at 1 year in our study. While urgent/emergent status was not a significant multivariable predictor of 1-year mortality, we may have been underpowered in this analysis. The increased risk of death at 1 year for the urgent/emergent TAVR population relative to the elective TAVR population is likely attributable to higher baseline risk with respect to a few select cardiac comorbidities. In similarity to the STS/ACC TVT registry findings, a higher proportion of patients undergoing urgent or emergent TAVR in our analysis carried pre-procedural diagnoses of heart failure, reduced left ventricular systolic function, and acute myocardial infarction or unstable angina. These are validated components of the Society of Thoracic Surgeons predicted risk of mortality score that convey higher baseline operative risk for both surgical aortic valve replacement as well as TAVR.^22^^,^^23^ There were also significant differences in aortic valve characteristics between groups, with patients undergoing urgent/emergent TAVR more likely to have bicuspid aortic valve anatomy or a prior aortic valve balloon valvuloplasty. Patients with bicuspid aortic valve anatomy account for 1 in 4 of all patients aged 80 years or older referred for aortic valve replacement and now make up approximately 10% of patients treated with TAVR.^24^ Prior studies of those with bicuspid anatomy undergoing TAVR have demonstrated comparable 1-year mortality rates relative to those with trileaflet valves yet higher rates of paravalvular leak, stroke, or annular rupture.^24^^,^^25^ It is possible concerns regarding these potential procedural complications may be contributing towards delayed treatment in the bicuspid aortic valve population and thus lower representation in the elective TAVR group. The high prevalence of chronic kidney disease, diabetes, anemia, and chronic lung disease across the acuity spectrum reflect the substantial burden of frailty and enhanced procedural risk in the general TAVR population.

We found that urgent or emergent TAVR status was an independent predictor of non-improved quality of life at 30 days relative to those who underwent elective TAVR. This finding did not persist at 1 year, however, although this may at least partially be due to survivorship bias because of the higher unadjusted rate of death over this time course in the urgent/emergent TAVR population. Major stroke and AKI following TAVR have previously been associated with poorer quality of life at 1 year in a PARTNER registry.^26^ While there was no difference in stroke or a composite of cardiovascular complications between groups in our study, we did observe that urgent/emergent TAVR status was also an independent predictor of hospitalized AKI within 1 year. This is possibly attributable to higher rates of HF hospitalization and vascular complications in this subgroup, both of which predispose to AKI. Overall, these findings suggest that procedure acuity status may be useful to identify and counsel patients that are less likely to experience short-term improvements in quality of life. It is important to note, however, that the majority of patients undergoing urgent or emergent TAVR in this study encountered acceptable or improved quality of life at 30 days and 1 year post-TAVR.

Our study has several limitations. First, while our inclusion of additional EHR data elements may mitigate the potential for unmeasured covariates relative to analyses restricted to STS/TVT registry data, this is an observational study that remains subject to residual and/or unmeasured confounding. Second, patients were stratified according to acuity documented by the treating team at the time of their procedure according to the STS/ACC TVT registry definitions, introducing the potential for intra- and interobserver variability and lack of accuracy in data entry/coding. Third, we used diagnosis code-based definitions for selected variables for this analysis, which is subject to potential recording errors. Fourth, although this study involved multiple centers, they are all part of our large, integrated health delivery system across a single geographic region so results may not be fully generalizable to other practice settings. Finally, our sample size limited our power to effectively investigate temporal trends, rare events, or to further stratify by acuity status (i.e., emergent vs. urgent).

In conclusion, in this community-based cohort of adults receiving TAVR, urgent or emergent status was uncommon and associated with high-risk clinical features and higher unadjusted rates of short- and long-term morbidity and mortality. Procedure acuity status may be useful to identify patients at risk for hospitalized AKI or that are less likely to experience significant short-term improvements in quality of life post-TAVR.

## Funding

This work was supported by Kaiser Permanente Northern California (KPNC), the authors’ academic institution, via a KPNC Community Benefit Grant. Andrew P. Ambrosy reports a relationship with National Heart Lung and Blood Institute that includes: funding grants; reports a relationship with Abbott Cardiovascular Structural Heart Division that includes: funding grants; reports a relationship with Edwards Lifesciences Corporation that includes: funding grants; reports a relationship with Esperion Therapeutics Inc that includes: funding grants; reports a relationship with Lexicon Pharmaceuticals, Inc. that includes: funding grants; reports a relationship with Novartis that includes: funding grants. Alan S. Go reports a relationship with National Heart Lung and Blood Institute that includes: funding grants; reports a relationship with National Institute of Diabetes and Digestive and Kidney Diseases that includes: funding grants; reports a relationship with National Institute on Aging that includes: funding grants; reports a relationship with Amarin Pharma Inc that includes: funding grants; reports a relationship with Novartis that includes: funding grants; reports a relationship with Janssen Research and Development LLC that includes: funding grants; reports a relationship with CSL Behring that includes: funding grants.

## Ethics Statement

This study was approved by the Kaiser Department of Research Institutional Review Board and followed institutional ethical guidelines.

## Disclosure Statement

Andrew P. Ambrosy is supported by a Mentored Patient-Oriented Research Career Development Award (K23HL150159) through the National Heart, Lung, and Blood Institute and has received research support through grants to his institution from Abbott, Amarin Pharma, Edwards Lifesciences, Esperion, Lexicon, and Novartis. Alan S. Go has received research support through grants to his institution from the National Heart, Lung and Blood Institute; National Institute of Diabetes, Digestive and Kidney Diseases; National Institute on Aging; Amarin Pharma, Inc.; Novartis; Janssen Research & Development; and CSL Behring. All other authors have no potential conflicts of interest to declare.
